# Effects of Dietary Vitamin A Concentration and Stress on Astaxanthin Utilization in Atlantic Salmon (*Salmo salar*)

**DOI:** 10.1155/anu/1961029

**Published:** 2026-07-14

**Authors:** Trine Ytrestøyl, Thea Morken, Tone-Kari Knutsdatter Østbye, Alexander Dikiy, Elena Shumilina, Marta Bou, Bjarne Hatlen, Gunvor Struksnæs, Anne Marie Langseter, Målfrid Bjerke, Julia Mullins, Aleksei Krasnov, Bente Ruyter

**Affiliations:** ^1^ Department of Aquaculture, Nofima, Sjølsengvegen 22, NO-6600, Sunndalsøra, Norway, nofima.no; ^2^ Skretting Aquaculture Innovation, Sjøhagen 6, NO-4016, Stavanger, Norway, skretting.com; ^3^ Department of Aquaculture, Nofima, Osloveien 1, NO-1433, ÅS, Norway, nofima.no; ^4^ Institute for Biotechnology and Food Science, NTNU, NO-7034, Trondheim, Norway, ntnu.no

**Keywords:** astaxanthin, Atlantic salmon, metabolism, stress, vitamin A

## Abstract

Diets with 30 and 60 ppm astaxanthin in combination with vitamin A (VA) concentrations of 6500, 35,000, and 100,000 IU/kg were fed to Atlantic salmon post smolts in seawater for 17 weeks. Flesh color (SalmoFan 27.1–27.5), astaxanthin concentration (3 mg/kg), retention (4.8% and 8.9% at 30 and 60 mg astaxanthin/kg respectively), and digestibility (29% and 12% at 30 and 60 mg astaxanthin/kg, respectively) were lowest at the highest dietary VA concentration. Increasing VA concentration in the diet also reduced the astaxanthin concentrations in liver and plasma, but not in intestine. Hepatocytes and enterocytes were isolated from fish fed the six diets and incubated for 48 h with radiolabeled astaxanthin to study metabolic transformation of astaxanthin in vitro. Astaxanthin was metabolized to idoxanthin, β‐carotene, retinol, and retinal in both cell types, but astaxanthin made up a larger proportion of total radioactivity in hepatocytes than in enterocytes. Hepatocytes from salmon fed high VA in the diet had the highest astaxanthin concentration (39% of the radioactivity), whereas the highest VA concentration (6% of the radioactivity) was found in hepatocytes from fish that received the lowest dietary VA concentration. Enterocytes from salmon fed the medium VA diet, had highest concentrations of astaxanthin (17%–26%), retinol (6.4%–7%) and the lowest concentration of idoxanthin (14%–31%). After the 17‐week feeding trial, salmon fed four of the diets (30 and 60 ppm astaxanthin in combination with 6500 and 100,000 IU/kg VA) were exposed to repeated stress (crowding and low oxygen) for a period of 5 weeks. Stress reduced the astaxanthin concentrations in muscle with around 0.5 mg/kg, except in salmon fed the diet with high VA and 60 ppm astaxanthin, which maintained an astaxanthin concentration of 3.3 mg/kg. Changes in the part of the carotenoid NMR spectrum in salmon exposed to stress indicated that stress affected the conversion of astaxanthin in interaction with dietary VA and astaxanthin. The effects of diet VA and astaxanthin concentration in combination with stress on gene expression in intestine, liver, and muscle were measured, and minor effects on several immune, stress, and metabolic genes were found.

## 1. Introduction

Fillet pigmentation is a key quality trait in farmed Atlantic salmon (*Salmo salar*), with the characteristic red color resulting from the deposition of the carotenoid astaxanthin in the muscle tissue. The efficiency of astaxanthin uptake and muscle retention is influenced by several factors, including dietary composition, inclusion level, life stage, growth rate, and environmental temperature [1–10]. Due to this, the retention of astaxanthin in muscle can vary considerably but is most often between 5% and 10% at dietary levels of around 40–50 mg/kg [3, 4, 7, 11, 12].

Recent observations suggest that there has been a decline in fillet color in farmed salmon over the past decade [13]. Increased oxidative stress has been proposed as a contributing factor, although the supporting experimental evidence is limited. Stress is unavoidable in aquaculture production, arising from fluctuating oxygen levels, handling, and elevated temperatures. High water temperatures, particularly during summer months in regions such as Tasmania, exacerbate stress in cold‐water species and have been linked to reduced astaxanthin content and fillet color [14, 15]. In Norway, water temperatures are not as high as in Tasmania, although summers are getting warmer, and some areas are above the optimum temperatures for growth. Warmer water increases problems with salmon lice infestations, and mechanical delousing treatments, which involve crowding and reduced oxygen concentrations, have become more frequent. A negative correlation between the number of mechanical delousing operations during a production cycle and fillet color has been shown in Norwegian salmon farming [13]. However, confounding effects such as reduced feed intake and growth may also contribute.

Stressful conditions increase the physiological demand for antioxidants, including astaxanthin, and vitamins. Astaxanthin is a potent antioxidant due to its conjugated double bond structure, which enables singlet oxygen quenching and free‐radical scavenging [16, 17]. It also modulates gene expression related to oxidative defense and cellular metabolism through its metabolites, apocarotenoids, and retinoids [18, 19]. Importantly, astaxanthin can be enzymatically converted into VA in Salmonids [20, 21], linking pigmentation directly to the VA status. While this conversion appears most active during early life stages [22, 23], its regulation in post‐smolt Atlantic salmon remains poorly understood. This metabolic link suggests that astaxanthin may contribute not only to pigmentation but also to VA‐dependent physiological functions, particularly under stress.

A major shift in salmon aquaculture has been the transition from marine‐based to plant‐based diets [24, 25], which has led to reduced levels of key nutrients such as phospholipids, omega‐3 fatty acids (EPA and DHA), cholesterol, and several vitamins, including VA [26]. The nutritional shift has also impacted the astaxanthin metabolism. Diets low in EPA and DHA have been associated with reduced fillet color and astaxanthin retention [27, 28], while diets high in marine ingredients may increase astaxanthin metabolism and reduce its deposition [29]. Several nutrients, including cholesterol and phospholipids, are known to influence astaxanthin absorption and conversion, further complicating the nutritional management of pigmentation. VA is an essential micronutrient with broad physiological roles, including antioxidant activity, immune modulation, and support for epithelial integrity [30]. Increased dietary supplementation has been shown to mitigate oxidative stress in terrestrial livestock [31], suggesting its potential relevance for aquaculture species exposed to production stress. In salmon, suboptimal VA levels, whether due to reduced dietary inclusion or impaired conversion from astaxanthin, may compromise the fish’s ability to cope with oxidative stress, potentially affecting both health and fillet pigmentation.

Understanding the interactions between dietary antioxidants, such as astaxanthin and VA, under stressful conditions is therefore essential for optimizing both fish health and product quality. Thus, the main objectives of this study were to assess the effect of different dietary concentrations of VA and astaxanthin on fillet color and astaxanthin retention and metabolism and to measure the effect of stress in interaction with diet on the fillet concentration of astaxanthin.

## 2. Methods

### 2.1. Experimental Design and Fish Husbandry

**Figure 1:**
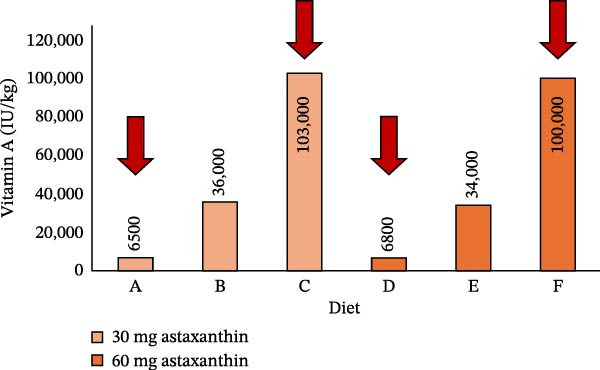
Combinations of astaxanthin and vitamin A in the six diets fed in the 17‐week trial. Red arrows indicate the four diets (A, C, D, and F) that were fed to a stressed group and a control group that was not stressed in the 5‐week elongation of the feeding trial.

The feeding experiment was carried out at Skretting Aquaculture Innovation (AI) Lerang Research station (Strand, Norway) and conducted according to the guidelines of the Norwegian State Commission for Laboratory Animals. The protocol was approved by the National Food Safety Authority (identification number: FOTS ID 24308). Triplicate groups of size‐graded Atlantic salmon (*Salmo salar*) with an initial average weight of 200 g were fed six experimental diets made by Skretting AI (Stavanger, Norway). The diets had three concentrations of VA (low ~6600 IU, medium ~35,000 IU, high ~100,000 IU) and astaxanthin concentrations of 30 or 60 mg/kg (Figure 1). The latter is close to the average astaxanthin concentration in Norwegian salmon diets [13]. The concentrations of VA were representative of the concentration that can be found in commercial salmon diets with different levels of marine ingredients. The basic formulation of the diets (A–F) was the same, and the inclusion level of marine ingredients was low to minimize the contribution of VA from marine ingredients and also to be commercially relevant (Table 1). A purified (deodorized) fish oil was used to ensure a low concentration of VA in the low VA diet. This diet design allowed the formulation of a basal diet with low background VA. An alternative approach would have required the use of semi‐purified diets, which are less representative of commercial salmon feeds. The mineral and vitamin premix used in the diets did not include VA, which was added separately to the experimental diets and was formulated to fulfill all other vitamin and mineral requirements for Atlantic salmon according to National Research Council [32]. Yttrium oxide was added to all diets as an inert indicator for the measurement of the apparent digestibility coefficient of astaxanthin (ADC). The chemical composition of the diets was analyzed by near‐infrared spectroscopy (NIR) by Skretting AI’s laboratory in Stavanger (Table 2). Astaxanthin and VA were analyzed by HPLC by Skretting AI and Masterlab (Boxmeer, the Netherlands), respectively (Table 2).

**Table 1:** Diet formulation.

Ingredient	Diet content
Fishmeal (%)	5.0
Wheat (%)	4.0
Wheat gluten (%)	22.0
Soy protein concentrate (%)	22.0
Pea protein concentrate (%)	10.0
Guar meal (%)	3.0–3.1
Fish oil (%)	11.0
Rapeseed oil (%)	13.8
Mineral and vitamin mix (%)	6.1
Carophyll pink (10% Astaxanthin)	0.03/0.06
Vitamin A‐1000	0/0.0035/0.0115
Yttrium oxide (%)	0.01


*Note:* Fishmeal: Prima Protein AS; wheat: Lantmännen Ek För; wheat gluten: Cargill PLC (Manchester); soy protein concentrate: European Commodity Company S.a.; pea protein concentrate: Yantai Shuangta Food Co., Ltd; guar meal: Sunita Hydrocolloids PVT. Ltd; fish oil: FF Skagen AS; rapeseed oil: Linas Agro Ab; Astaxanthin and vitamin A: DSM‐Firmenich; mineral, vitamin and yttrium oxide premixes: Trouw Nutrition. Mineral and vitamin mix: Skretting.

**Table 2:** Chemical composition of experimental diets.

Diet content	A 30/L	B 30/M	C 30/H	D 60/L	E 60/M	F 60/H
Moisture (%)	7.6	7.6	7.8	7.6	7.7	8.1
Ash (%)	4.0	3.8	4.0	4.2	4.0	4.0
Protein (%)	45.8	46.4	45.7	45.8	45.8	46.1
Fat (%)	30.4	30.6	30.4	30.6	30.4	30.3
Astaxanthin (mg/kg)	29.8	30.7	30.0	59.1	58.8	59.4
Vitamin A (IU)	6500	36,000	103,000	6800	34,000	100,000

Abbreviations: 30, 30 ppm astaxantin; 60, 60 ppm astaxanthin; H, high vitamin A; L, low vitamin A; M, medium vitamin A.

Prior to the trial, the fish had been smoltified using a period of 7 weeks with short days (12 h light and 12 h darkness, 353 day degrees) followed by 5 weeks on 24 h light (418 day degrees) before transfer to seawater. Fish were acclimatized in the experimental tanks for 14 days, weighed, and sorted before starting the trial. The weight of the fish when the trial started was 190 ± 2 g. During the trial, the fish were maintained indoors in 0.45 m^3^ tanks (35 fish per tank) on continuous light and supplied with seawater (32 ppt) with an average temperature of 12°C. Each diet was fed to triplicate tanks for a period of 17 weeks. The fish were fed by automatic feeders three times per day. The tank outlets were passed through an excess feed collector, which enabled calculation of accurate feed intake on the basis of dry matter (DM) analyses of the collected feed. This provided data for estimation of astaxanthin retention in the muscle (in % of the eaten amount of astaxanthin). After 17 weeks, the feeding trial was terminated, and samples of fillet, blood, liver, and intestine were taken from 10 fish per tank as described in Section 2.2.

Fish fed diets with high and low VA content combined with 30 and 60 ppm astaxanthin (diets A, C, D, and F) were then redistributed into 16 larger tanks (7 m^3^, 25 fish per tank) and were fed these four diets in duplicate for a period of 5 weeks. There was a limited number of available tanks, so all diets could not be tested in triplicate in the stress trial. The high and low VA and astaxanthin levels were chosen for the stress protocol because they were suspected to give the largest differences in response to the stress protocol. Fish in half of the tanks were subjected to stress by lowering the water and oxygen levels in the tanks three times per week. The water flow into the tanks was reduced until the water level reached 30 cm from the bottom of the tank over a period of 10 min. The water level was kept at this level until the oxygen saturation in the tanks was reduced to 35%, which is considered the limiting oxygen saturation at 12°C for post‐smolt Atlantic salmon [33]. Fish in the control group were not exposed to stress. The fish were weighed at the start and at the end of the 5‐week stress challenge period, and muscle samples were taken for the analysis of carotenoids and metabolites of astaxanthin after the trial was finished. The samples were analyzed by HPLC to determine astaxanthin and idoxanthin in the fillet.

### 2.2. Collection of Samples

After 17 weeks of feeding the experimental diets, samples of blood, liver, mid‐intestine, and fillet were taken from 10 fish per tank. Feces were collected by stripping the remaining fish in the tank according to [34]. The fish were anesthetized with MS‐222 (metacaine, 0.1 g L^−1^) before length and weight were recorded. The fish were killed by a blow to the head, and samples of muscle, liver, and mid‐intestine were collected for microarray analysis from five fish per tank. The middle section of the mid‐intestine and liver were dissected out, and small pieces (1 × 2 mm) were frozen immediately in liquid nitrogen and kept at −80°C until analysis. Muscle samples were taken 2 cm above the lateral line on the Norwegian quality cut (NQC) section. Blood samples were taken from the caudal vein of six fish per tank. The samples were centrifuged (5000 g, 10 min), and the plasma was frozen at −80°C for later analysis of carotenoids. Fillets (right side) from 10 individual fish were sampled per tank, and visual color was evaluated as described below. The fillets were thereafter skinned and homogenized individually before analyzing fat and astaxanthin in the muscle using NIR (Skretting AI). Astaxanthin and idoxanthin were analyzed in the muscle, liver, intestine, and blood by HPLC in pooled samples from six fish per tank. Feces were analyzed for astaxanthin content, and the apparent digestibility coefficients of astaxanthin (ADC) were calculated for the six diets.

At the end of the stress challenge trial, fillets (right side) from 10 individual fish were sampled per tank for the analysis of astaxanthin concentration. Samples for microarray analysis of the mid‐intestine, liver, and muscle were taken as described above from five fish per tank fed diets with 60 ppm astaxanthin in combination with high or low vitamin A concentrations.

### 2.3. Isolation of Hepatocytes and Enterocytes and Incubation With ^14^C Astaxanthin

At the end of the feeding trial, liver and intestinal cells were isolated from salmon in all six diet groups. The midgut was removed from the salmon, cut open, and washed several times in DPBS. The tissue was then transferred to DPBS with 30 mM EDTA and 1.5 mM DTT before rinsing in L‐15. To obtain a representative sample of the intestinal epithelium, the luminal surface of the intestine was gently scraped using a sterile glass slide. This procedure collects the entire cellular layer lining the mucosa, including enterocytes and other epithelial cell types. The collected material was transferred to L‐15 and subsequently processed to isolate the enterocytes. The material was centrifuged at 377 g and 4°C for 5 min. The pellet was resuspended in 0.1% collagenase solution (0.14M NaCl, 7 mM KCl, 0.01M HEPES, 0.1% collagenase, 1.5 mM CaCl_2_, pH 7.4) and incubated at 13°C for 30 min. The cell suspension was filtered through a 250 μm and then a 100 μm filter to separate cell debris from enterocytes before centrifugation at 377 g and 4°C for 5 min. The enterocyte cell suspension was then washed a couple of times in growth medium (10% FBS, 1× AntiAnti, 0.01M HEPES, and 0.04% NaHCO_3_) and resuspended in growth medium with 2% FBS. 1 × 10^7^ cells were added to T25 cell flasks and 5 x 10^4^ cells per well in 96‐well plates. Liver cells were isolated according to Kjær et al. [35]. Liver and intestinal cells were cultured at 13°C in growth medium supplemented with 0.9 μg/mL radioactive astaxanthin at 13°C for 48 h. Subsequently, radioactive astaxanthin, VA, and other metabolites of astaxanthin were determined using UPLC (Acquity H‐class plus, Waters Corporation) connected to a radio detector (FlowStar LB 513, Berthold Technologies).

### 2.4. Analyses

#### 2.4.1. Visual Flesh Color

**Figure 2:**
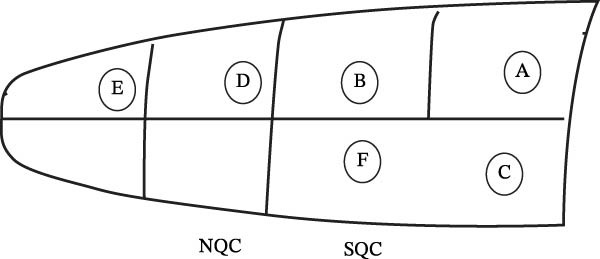
Positions for Minolta measurements (A–F) and registrations with SalmoFan Color scale (B, D, and F).

Visual flesh color was evaluated by two observers trained in the use of SalmoFan (DSM, scale 20–34) and also by using a Minolta Chroma Meter (CR‐400 Minolta, Konica Minolta Sensing Inc., Japan) on six positions on the fillet (Figure 2). In the CIE 1976 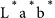 color space (also referred to as CIELAB), 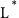 indicates lightness and 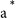 and 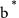 are chromaticity coordinates. 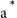 and 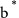 are color directions: 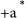 is the red axis and 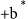 is the yellow axis.

#### 2.4.2. Measurement of Astaxanthin Concentration (NIR and HPLC)

Astaxanthin was analyzed in homogenized samples of fillet using near‐infrared spectroscopy (NIR; NIR Systems XDS Rapid Content Analyzer, Metrohm Nordic AS, Oslo, Norway) connected to an ISIscan instrument with the WinISI software package (Foss) using equations developed by Skretting AI based on the HPLC method described by Schierle et al. [36].

Carotenoids in the muscle, intestine, and liver were analyzed as described in Bjerkeng et al. [37]. Carotenoids were extracted with chloroform, and the extract was evaporated to dryness and then dissolved in a mobile phase consisting of 20% acetone in n‐hexane. A Spherisorb S5‐CN nitrile column (250 × 46 mm) was used to determine the cis and trans isomers of astaxanthin and idoxanthin. Standards with known concentrations were prepared from crystalline all‐E‐astaxanthin (Hoffmann‐La Roche Ltd., Basel, Switzerland), and the concentration of the standard solution was measured spectrophotometrically (UV‐260, Shimadzu, Japan) using molar absorptivity E1%, 1 cm = 2100 at absorbance maximum (λ_max_ = 470 nm) in n‐hexane with 4.5% chloroform. The percentage of the different isomers was calculated from chromatogram areas and corrected for differences in extinction coefficients (E1%, 1 cm) [38–40]. Astaxanthin in feed and feces was extracted according to the procedure described by Schierle and Härdi [41]. An H_3_PO_4_‐modified silica gel column (Hibar, LiChrosorb Si 60; Merck, Darmstadt, Germany) was then used to quantify the E/Z isomers of astaxanthin in feed and feces.

The astaxanthin concentrations of the feeds and fecal samples were analyzed following the extraction of the carotenoids according to the procedure described by Schierle et al. [42]. Homogenized fillets from individual fish were thawed, and carotenoids were analyzed according to Bjerkeng et al. [37]. Two isocratic HPLC systems were used to determine carotenoid concentrations; a H_3_PO_4_‐modified silica gel column (Hibar, LiChrosorb Si 60; Merck, Darmstadt, Germany) was used to determine the geometrical E/Z isomers of astaxanthin in feed and feces. In muscle, liver, and intestine, a Spherisorb S5‐CN nitrile column (PhaseSep, Queensferry, Clywd, UK) was used with 20% acetone in n‐hexane as the mobile phase to determine astaxanthin and 3′, 4′‐cis and 3′, 4′‐trans glycolic isomers of idoxanthin. Standards of known concentration were prepared from crystalline all‐E‐astaxanthin (Hoffmann‐La Roche Ltd., Basel, Switzerland), and the concentration of the standard solution was measured spectrophotometrically (UV‐260, Shimadzu, Japan) using molar absorptivity E1%, 1 cm = 2100 at absorbance maximum (λ_max_ = 470 nm) in n‐hexane containing 4.5% chloroform. The percentages of the different isomers were calculated from chromatogram areas and corrected for differences in extinction coefficients (E1%, 1 cm) [38–40].

#### 2.4.3. NMR

The muscles of salmon fed four different diets were analyzed: Diet A (low astaxanthin, low retinol), Diet C (low astaxanthin, high VA), Diet D (high astaxanthin, low VA), and Diet F (high astaxanthin, high VA). Muscle samples were extracted using a modified protocol based on that of Shumilina et al. [43]. In brief, 5 g of the salmon muscle homogenate was extracted with 15 mL of acetone. After the removal of water and solvent, the solid residue was dissolved in 0.6 mL of deuterated acetone containing TMS as an NMR standard.

1D ^1^H NMR spectra of acetone extracts were acquired at 300 K on a Bruker Avance 600 MHz spectrometer equipped with a 5 mm z‐gradient TXI (H/C/N) cryoprobe using water pre‐saturation experiments (Bruker pulse sequence noesygppr1d) with the following parameters: d1 4 s; SW 20 ppm; NS 256; and RG 9.

Topspin 4.0.7 software (Bruker, Germany) was used for spectral calibration and baseline correction.

The carotenoid region (6.682–5.864 ppm), where resonances of conjugated double bonds typical of carotenoids are found, was processed using AMIX software (Bruker, Germany). This region was segmented into 824 uniform integration intervals (“buckets”) of 0.001 ppm width. Each fish was thus characterized by its own set of 824 integral values, with each integral representing the relative concentration of carotenoids at that chemical shift.

This set of integrals, describing the carotenoid profile of each fish, was used as input for ANOVA (SPSS software) to identify variables (i.e., spectral regions) that were statistically different between groups. For fish (samples) from the control experiment, 123 significant variables were identified. The same workflow applied to stressed fish resulted in 221 significant variables. Following identification of significant variables, linear discriminant analysis (LDA) was performed to explore diet‐ and stress‐related patterns.

To investigate individual metabolic variation within each diet/stress, spectra were visually inspected to identify outliers, defined as spectra with abnormal peak shapes or nonconforming baseline features in the carotenoid region. After the removal of outliers, the ANOVA–LDA workflow was repeated, yielding 77 significant variables for control samples and 202 for stressed samples.

#### 2.4.4. Microarray

RNA was extracted with a Biomek 4000 robot using the Agencourt RNAdvance Tissue kit (Beckman Coulter), and quality was assessed with an Agilent Bioanalyzer 2100, RNA 6000 nano kit (RIN > 8). Nofima’s genome‐wide Atlantic salmon microarray Salgeno with 44 k 60‐mer oligonucleotide probes was used. The platform was annotated with the bioinformatics package STARS [44]. Microarrays were manufactured by Agilent Technologies, and the reagents and equipment were purchased from the same provider. RNA amplification and labeling were performed with a One‐Color Quick Amp Labeling Kit, and a Gene Expression Hybridization kit was used for fragmentation of labeled RNA. The total RNA input for each reaction was 500 ng. After overnight hybridization in an oven (17 h, 65°C, rotation speed 0.01 g), arrays were washed with Gene Expression Wash Buffers 1 and 2 and scanned with an Agilent scanner.

#### 2.4.5. Sample Preparation for Retinoid and Carotenoid Analysis From In Vitro Study

The cell material was transferred to Eppendorf tubes for extraction. Retinoid and retinyl ester analyses were performed following protocols adapted from Blomhoff et al. [45, 46]. Briefly, 0.1–0.5 mL of cell homogenate was mixed with 1 mL of methanol containing 0.1% butylated hydroxytoluene (BHT), vortexed thoroughly, and sonicated to ensure complete cell disruption and stabilization of lipophilic compounds.

From the homogenate, 100 µL was aliquoted into a separate vial for subsequent determination of the total radioactivity and protein content. The remaining sample was divided equally into two portions (0.45 mL each) and transferred to individual vials designated for carotenoid and retinoid extraction, respectively. This division allowed the parallel processing and quantification of both compound classes under optimized conditions.

#### 2.4.6. Carotenoid Extraction

For carotenoid extraction, 0.45 mL of methanol containing 0.1% BHT was added to the sample vial to stabilize carotenoids and prevent oxidation. The mixture was then combined with 1.8 mL of chloroform and 0.45 mL of distilled water, vortexed thoroughly, and centrifuged at 3000 rpm for 10 min to achieve phase separation. The chloroform phase, containing the extracted carotenoids, was carefully transferred to a light‐protected glass vial. The solvent was evaporated under a gentle stream of nitrogen at room temperature. The resulting residue was reconstituted in methanol with 0.1% BHT, flushed with nitrogen to minimize oxidation, and stored at −80°C until analysis.

#### 2.4.7. Retinoid Extraction

For retinoid analysis, 0.45 mL of the homogenized sample was transferred to glass tubes and subjected to saponification to hydrolyze retinyl esters. Hydrolysis was performed by adding 3 mL of 20% potassium hydroxide (KOH) in ethanol containing 1% pyrogallol, an antioxidant used to prevent the oxidative degradation of retinoids. The reaction mixture was incubated at 57°C for 30 min in a heat block and protected from light throughout the procedure.

Following hydrolysis, 2.5 mL of hexane containing 0.1% BHT was added to extract free retinol. The mixture was vortexed thoroughly, and the hexane phase was carefully separated and transferred to a clean glass vial. The extract was then filtered through a 0.2 µm membrane to remove particulates and evaporated under a gentle stream of nitrogen at 30°C.

The resulting residue was reconstituted in methanol containing 0.1% BHT, flushed with nitrogen to minimize oxidation, and stored in amber vials at −80°C until the analysis.

#### 2.4.8. Preparative Thin‐Layer Chromatography (Prep‐TLC)

Prep‐TLC was performed using pre‐washed HPTLC plates (20 × 10 cm), which were run vertically to optimize compound separation. Samples were applied manually using a Hamilton syringe. After application, the sample area was lightly covered to protect it from light and environmental exposure. Standards of retinol, retinal, β‐caroten, and astaxanthin were also applied to the plate for reference.

The mobile phase consisted of a mixture of acetone and heptane (30:70, v/v). Chromatography was conducted under light‐protected conditions using opaque coverings to minimize photo‐degradation of sensitive compounds.

Following the development, the plate was first photographed under UV light to visualize fluorescent compounds. It was then dipped in a copper sulfate (CuSO_4_) solution for chemical development and subsequently photographed under visible‐light wavelengths immediately after development to capture band resolution.

Target bands of radiolabelled retinol, retinal, β‐carotene, and astaxanthin were scraped from the plate and transferred to scintillation vials. Each sample was treated with a single addition of Flo‐Scint scintillation fluid for radioactivity counting in a scintillation counter. The proportion of radioactively labeled individual molecules was calculated as a percentage of the total radioactivity measured in the sample.

### 2.5. Calculations and Statistical Analysis

The specific growth rate (% day^−1^) was calculated as:

SGR=ln BW2 −ln BW1×100/dBW= bodyweight,d=number of days.

BW_1_ and BW_2_ are the body weights at the start and end of the feeding period, respectively.

The thermal growth coefficient, TGC, was calculated as follows:

TGC =1000×BW213/ − BW113/×number of day degrees−1.

The feed conversion ratio (FCR) was calculated as feed eaten (kg)/weight gain (kg).

Apparent digestibility ADC=100×1− Xfaeces/Yfaeces/ Xfeed/Yfeed .

*X* = concentration (mg kg^−1^) of astaxanthin/fat, and *Y* = concentration of yttrium oxide.

The retention of astaxanthin in the muscle was calculated as:

Retention %=100×Wmu/BW×BW2×Ax2−BW1×Ax1 /FI×FCR×Axfeed,

where AX_1_ and AX_2_ are the concentrations of astaxanthin in the fillet at the start and end of the experiment, respectively, BW_1_ and BW_2_ are the body weights at the start and end, and *W*_mu_ is the weight of the muscle, which was assumed to constitute 60% of the total body weight in Atlantic salmon. FI is the total amount of feed consumed (g per fish) and FCR is the feed conversion ratio (g feed consumed/g weight gain).

Statistical analysis on growth, bodyweight FCR, fillet color, and NIR data were performed in GraphPad Prism (version 10.3.0) using 2‐way ANOVA with the dietary content of vitamin A and astaxanthin as fixed factors. Statistical analyses on carotenoid concentrations in muscle, blood, intestine, and liver and effects of stress were done in SAS Jmp and GraphPad Prism (2‐way and 3‐way ANOVA with dietary astaxanthin and retinol concentrations and stress as fixed factors, respectively). Post‐hoc analyses (Tukey’s multiple range test) were done to rank different treatments. *p*‐values < 0.05 were considered significant. Response variables given in percent were arcsin transformed before analysis by ANOVA. Correlation between variables was done in SAS Jmp.

Analyses of microarray data were carried out with the STARS. Global normalization was performed by equalizing the mean intensities of all microarrays. Next, the individual values for each feature were divided to the mean value of all samples producing expression ratios (ER). The log2‐ER was calculated and normalized with the locally weighted nonlinear regression (Lowess). Differentially expressed genes (DEG) were selected by criteria: ER >1.75‐fold and *p*  < 0.05.  

To determine significant metabolic variations for the NMR data, SPSS statistical software was used for variable selection, followed by Principal Component Analysis (PCA) and LDA using SPSS and Unscrambler software.

## 3. Results

### 3.1. Feeding Trial

#### 3.1.1. Growth and Feed Utilization

There were no significant effects of diet on feed intake, growth, and FCR (Table 3). There was, however, a tendency for interactions between dietary astaxanthin and VA concentration on feed intake (*p* = 0.07) and FCR (*p* = 0.05). Fish fed a combination of high dietary astaxanthin and VA had lower feed intake than fish fed with high VA and low astaxanthin.

**Table 3:** Body weight (BW, g) condition factor (CF), feed intake (FI, %/day), feed conversion factor (FCR), specific growth rate (SGR), and thermal growth factor (TGC) of Atlantic salmon fed the six experimental diets for 17 weeks.

Performance metrics	Diet A 30/L	Diet B 30/M	Diet C 30/H	Diet D 60/L	Diet E 60/M	Diet F 60/H	P Ax	P VA	Interaction Ax ^∗^VA
BW	972 ± 21	1012 ± 17	1018 ± 13	1008 ± 30	1004 ± 14	967 ± 14	0.65	0.54	0.06
CF	1.47 ± 0.02	1.47 ± 0.01	1.48 ± 0.01	1.47 ± 0.03	1.47 ± 0.01	1.45 ± 0.01	0.07	0.43	0.12
FI	1.09 ± 0.02	1.11 ± 0.02	1.13 ± 0.01	1.10 ± +0.02	1.12 ± 0.02	1.08 ± 0.01	0.39	0.52	0.07
FCR	0.79 ± 0.00	0.80 ± 0.01	0.81 ± 0.01	0.80 ± 0.00	0.81 ± 0.01	0.80 ± 0.01	0.73	0.12	0.05
SGR	1.37 ± 0.02	1.39 ± 0.01	1.39 ± 0.01	1.38 ± 0.03	1.38 ± 0.01	1.36 ± 0.01	0.39	0.71	0.29
TGC	2.88 ± 0.05	2.95 ± 0.03	2.96 ± 0.02	2.94 ± 0.07	2.93 ± 0.03	2.86 ± 0.03	0.48	0.64	0.14

*Note:* Values are shown as mean values using tank as a statistical unit (*n* = 3) with their standard error mean.

Abbreviations: 30, 30 ppm astaxantin; 60, 60 ppm astaxanthin; Ax, astaxanthin; H, high vitamin A; L, low vitamin A; M, medium vitamin A; VA, vitamin A.

#### 3.1.2. Flesh Color and Astaxanthin Deposition

**Figure 3:**
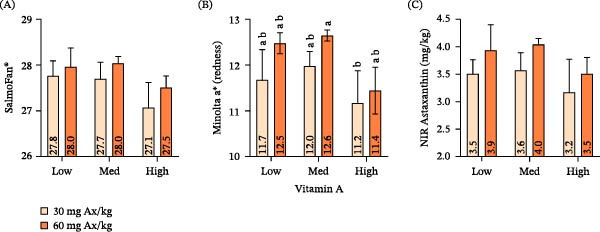
SalmoFan scores (A), Minolta a^∗^ values (redness) (B), and NIR astaxanthin (mg/kg) in fillet (C) from salmon fed diets with different combinations of astaxanthin (Ax) and VA concentrations for 17 weeks. Values are means per treatment ±SEM (*N* = 3).

**Figure 4:**
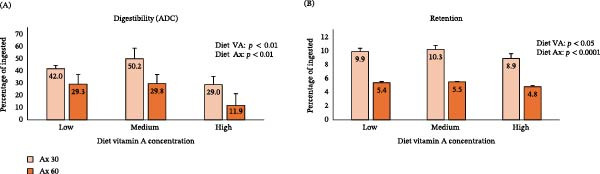
(A) Apparent digestibility (ADC) and (B) Muscle retention of astaxanthin in *A. salmon* fed six diets with different concentrations of vitamin A (low, medium, high) and two concentrations of astaxanthin (30 or 60 mg/kg) for 17 weeks. Values are mean ± SEM (*N* = 3).

The visual flesh color was affected by both dietary astaxanthin and VA concentrations. The SalmoFan score and Minolta 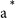 values (redness) were lower for fish fed diets with high VA concentration (Figure 3). The flesh astaxanthin concentrations obtained by NIR and HPLC confirmed the results on visual flesh color, with a negative effect of the highest dietary VA concentration on the fillet astaxanthin concentration independent of diet astaxanthin concentration (Table 4). The muscle retention of astaxanthin (% of eaten astaxanthin) was lower for salmon fed the diet with 60 mg of astaxanthin per kg compared to salmon fed diets with 30 mg of astaxanthin per kg. The retention was lower at the highest dietary VA concentration irrespective of diet astaxanthin concentration (Figure 4).

**Table 4:** Astaxanthin (Ax) idoxanthin and total carotenoid concentrations (mg/kg) in the muscle from salmon fed diets with different combinations of astaxanthin and vitamin A (VA) for 17 weeks.

	Diet A 30/L	Diet B 30/M	Diet C 30/H	Diet D 60/L	Diet E 60/M	Diet F 60/H	*p*‐Value VA	*p*‐Value Ax	Interaction VA × Ax
Astaxanthin	3.2 ± 0.2^a^	3.3 ± 0.2^a^	3.0 ± 0.3^b^	3.5 ± 0.1^A^	3.6 ± 0.0^A^	3.1 ± 0.1^B^	0.05	0.10	0.88
Idoxanthin	0.3 ± 0.0	0.2 ± 0.0	0.2 ± 0.0	0.4 ± 0.0	0.4 ± 0.1	0.3 ± 0.0	0.27	0.04	0.50
Total carotenoids	3.5 ± 0.3^a^	3.6 ± 0.4^a^	3.2 ± 0.4^b^	3.9 ± 0.3^AB^	4.0 ± 0.1^A^	3.4 ± 0.2^B^	0.04	0.04	0.76
Idoxanthin (%)	8.6 ± 0.4	6.8 ± 0.8	7.4 ± 1.2	9.5 ± 0.8	9.9 ± 2.2	8.3 ± 1.1	0.63	0.13	0.59

*Note:* Values are mean ± SEM (*N* = 3). Different superscript lowercase and uppercase letters indicate statistically significant differences (*p* < 0.05).

Abbreviations: 30, 30 ppm astaxantin; 60, 60 ppm astaxanthin; Ax, astaxanthin; H, high vitamin A; L, low vitamin A; M, medium vitamin A; VA, vitamin A.

#### 3.1.3. Carotenoid Concentration in Plasma, Liver, and Intestine

**Figure 5:**
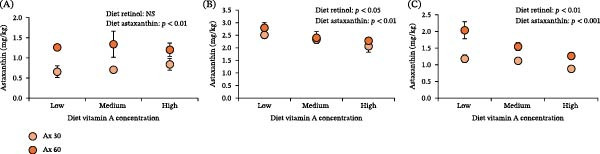
Astaxanthin concentrations in mid‐intestine, plasma, and liver in *A. salmon* fed six diets with different concentrations of vitamin A (low, medium, high) and two concentrations of astaxanthin (30 or 60 mg/kg) for 17 weeks. Values are mean ± SEM (*N* = 3). (A) Mid‐intestine. (B) Plasma. (C) Liver.

A higher dietary astaxanthin concentration increased the astaxanthin concentration in the mid‐intestine (Figure 5A), but there was no significant effect of dietary VA level. No significant correlations were found between the astaxanthin concentration in the intestine and the concentrations in muscle, plasma, and liver. Diet had no effect on the concentration of the astaxanthin metabolite idoxanthin in the intestine (Table S1). Plasma astaxanthin concentration decreased linearly with increasing dietary VA concentration, and there was a positive effect of higher dietary astaxanthin (Figure 5B). There were no diet effects on the plasma idoxanthin concentration (Table S1). A higher dietary astaxanthin concentration increased the liver astaxanthin concentration, whereas higher dietary VA concentrations reduced the liver astaxanthin concentration (Figure 5C). Salmon fed low dietary VA had higher idoxanthin concentration in liver than salmon fed high dietary VA concentration (*p*  < 0.05, S1). A strong positive linear correlation was found between liver and plasma astaxanthin concentrations (*p*  < 0.0001, *R* ^2^ = 0.70) and a weaker positive correlation was found between liver and muscle astaxanthin concentrations (*p*  < 0.01, *R* ^2^ = 0.36).

#### 3.1.4. Metabolism of Radiolabeled Astaxanthin in Cultures of Enterocytes and Hepatocytes

In both enterocytes and hepatocytes, a large part of the total recovered radioactivity on the TLC plates was found at the application point (on average 51% and 39% of the total radioactivity, respectively). These compounds were not identified. Overall, the diet had larger effects on the metabolism of astaxanthin in enterocytes than in hepatocytes, and it was the VA content in the diet that had the largest impact on the content of metabolites.

**Figure 6:**
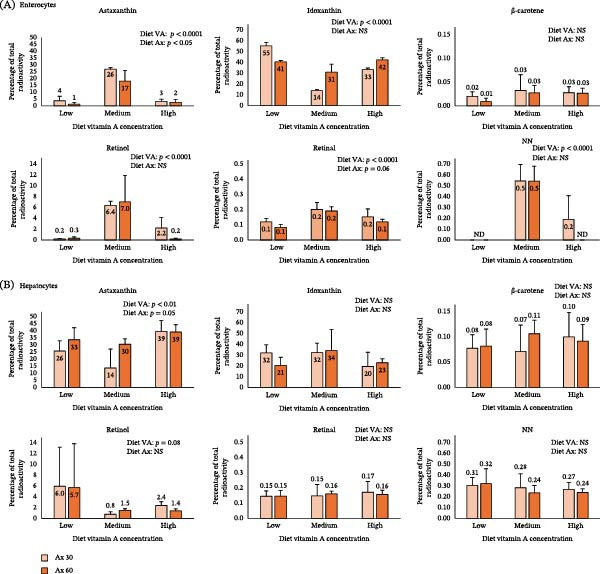
Concentration of astaxanthin and metabolites of astaxanthin expressed as % of total radioactivity in (A) enterocytes and (B) hepatocytes isolated from salmon fed six diets with low, medium, and high concentrations of vitamin A (VA) and 30 and 60 ppm of astaxanthin (Ax) for 17 weeks. Values are mean ± SD (*N* = 3). NN = unidentified astaxanthin metabolite.

In enterocytes, the medium VA diet gave a higher concentration of the radioactive astaxanthin substrate compared to the high and low VA diets, whereas higher diet astaxanthin concentration had a negative effect on the content of radioactive astaxanthin (Figure 6A). Idoxanthin was the most abundant radiolabeled metabolite identified in enterocytes. Depending on the diet, between 14% and 55% of the total radioactivity recovered in the enterocytes was idoxanthin (Figure 6A). Low dietary VA gave the highest idoxanthin concentration, and the medium VA diet gave the lowest idoxanthin concentration. Diet astaxanthin concentration did not affect the idoxanthin concentration in enterocytes. The dietary VA concentration also affected the amount of radioactive retinol (vitamin A1) and retinal in the enterocytes (Figure 6A). The highest concentrations were found in enterocytes from fish‐fed diets with medium VA concentrations. This was also the case for an unidentified metabolite (NN, Figure 6A). The concentrations of β‐carotene in the enterocytes were low (0.01%–0.03% of total radioactivity) and were not affected by diet astaxanthin or VA concentrations. However, there was a strong positive correlation between the concentration of β‐carotene and retinal (*p*  < 0.0001, *R* ^2^ = 0.80) and a weaker correlation between β‐carotene and idoxanthin (*p*  < 0.05, *R* ^2^ = 0.22). There were also weak positive correlations between retinal and retinol (*p*  < 0.01, *R* ^2^ = 0.27) and between retinal and idoxanthin (*p*  < 0.01, *R* ^2^ = 0.30).

Astaxanthin made up a larger proportion of the total recovered radioactivity in hepatocytes compared to in the enterocytes (*p*  < 0.01). The high VA diet gave a higher astaxanthin concentration in the hepatocytes (Figure 6B), and a high astaxanthin concentration in the diet also tended to increase the astaxanthin concentration of the hepatocytes. As in the enterocytes, idoxanthin was the most abundant radiolabeled metabolite identified in the hepatocytes, but diet did not affect the idoxanthin concentration (Figure 6B). There was also no significant effect of diet on the content of radioactive retinol, retinal, NN, or β‐carotene in the hepatocytes. There was, however, a tendency for a higher retinol concentration in hepatocytes from fish fed the low VA diet (*p* = 0.08), but the individual variation was quite large. The content of β‐carotene was higher in hepatocytes than in the enterocytes (*p*  < 0.01). There was a strong positive correlation between the concentrations of astaxanthin and β‐carotene (*p*  < 0.0001, *R* ^2^ = 0.80) and between β‐carotene and retinal (*p*  < 0.0001, *R* ^2^ = 0.87). Significant positive correlations were also found between astaxanthin and idoxanthin (*p*  < 0.01, *R* ^2^ = 0.30), β‐carotene and idoxanthin (*p*  < 0.001, *R* ^2^ = 0.44), and between idoxanthin and retinal (*p*  < 0.001, *R* ^2^ = 0.42).

### 3.2. Stress Challenge Trial

#### 3.2.1. Growth and Feed Utilization

There was no mortality during the stress challenge trial, and stress did not affect the feed intake, final weight, or feed utilization of fish (Table 5). The mean weight of the control group was 1259 ± 67 g and the stressed group weighed on average 1245 ± 78 g. There were no significant effects of diet astaxanthin or VA concentration on growth rate, but there was a tendency for an interaction between treatment (stress/control) and dietary VA concentration on growth (*p* = 0.098) so that stressed fish fed a high VA diet grew less than control fish on a high VA diet (Table 5).

**Table 5:** Body weight (BW), condition factor (CF), feed conversion ratio (FCR), specific growth rate (SGR), and thermal growth coefficient (TGC) of Atlantic salmon during the 5‐week stress‐challenge phase fed four selected diets (A, C, D, F).

Treatment	Diet	Ax (mg/kg)	VA	SGR (%/day)	TGC	FI (%/day)	FCR	BW_final_ (g)	CF
Control	A	30	Low	0.63 ± 0.05	1.79 ± 0.11	0.70 ± 0.03	1.12 ± 0,03	1258 ± 31	1.41 ± 0.02
	C	30	High	0.67 ± 0.01	1.94 ± 0.02	0.70 ± 0.02	1.04 ± 0,04	1316 ± 11	1.44 ± 0.02
	D	60	Low	0.65 ± 0.03	1.84 ± 0.10	0.72 ± 0.01	1.11 ± 0,03	1241 ± 68	1.40 ± 0.01
	F	60	High	0.74 ± 0.15	2.08 ± 0.45	0.71 ± 0.05	1.00 ± 0,15	1222 ± 73	1.40 ± 0.02
Stress	A	30	Low	0.75 ± 0.14	2.13 ± 0.42	0.74 ± 0.01	1.01 ± 0,19	1228 ± 74	1.42 ± 0.01
	C	30	High	0.51 ± 0.01	1.47 ± 0.03	0.60 ± 0.01	1.17 ± 0,03	1245 ± 48	1.40 ± 0.02
	D	60	Low	0.65 ± 0.10	1.90 ± 0.27	0.62 ± 0.07	0.96 ± 0.04	1331 ± 6	1.42 ± 0.01
	F	60	High	0.59 ± 0.04	1.65 ± 0.11	0.63 ± 0.04	1.07 ± 0.01	1173 ± 19	1.40 ± 0.02

*Note:* Half of the tanks underwent thrice‐weekly low‐water/low‐oxygen challenges and half served as non‐stressed controls. Values are mean ± SEM (*n* = 3) tanks per treatment.

#### 3.2.2. Muscle Carotenoid Concentrations

**Figure 7:**
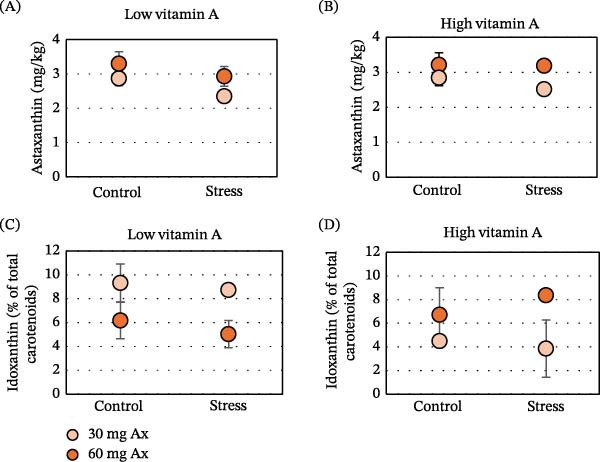
Muscle astaxanthin (A, B) and idoxanthin (C, D) of Atlantic salmon after the 5‐week stress‐challenge phase fed four selected diets (A, C, D, F). Half of the tanks underwent thrice‐weekly low‐water/low‐oxygen challenges and half served as non‐stressed controls. Values are mean ± SEM. *N* = 2 tanks per treatment.

**Figure 8:**
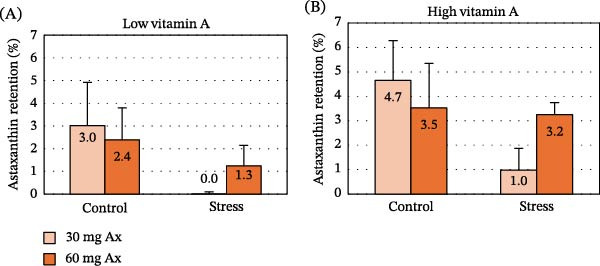
Muscle retention of astaxanthin (% of eaten) of Atlantic salmon after the 5‐week stress‐challenge phase fed four selected diets (A, C, D, F). (A) Diets with low VA, (B) diets with high VA. Half of the tanks underwent thrice‐weekly low‐water/low‐oxygen challenges and half served as non‐stressed controls. Values are mean ± SEM. *N* = 2 tanks per treatment.

There was no correlation between individual fish weight and the fillet astaxanthin concentration. A higher dietary astaxanthin concentration had a positive effect on flesh astaxanthin concentration both in stressed salmon and in control groups (*p*  < 0.01). Stress reduced the muscle astaxanthin concentrations by 0.4–0.6 mg/kg in three of the four diet treatments (*p*  < 0.05). The exception was in salmon fed the diet with high dietary astaxanthin and VA concentrations (Figure 7A,B). Due to the lower muscle astaxanthin concentrations, the astaxanthin retention in the muscle was also lower in stressed salmon (*p*  < 0.05, Figure 8). The average retention in the control group was 3.4%, whereas in stressed salmon, the average retention was only 1.4%. However, stressed salmon fed the low VA/low Ax diet had close to 0% retention of astaxanthin, while the non‐stressed control group fed the same diet had 3% retention (Figure 8A). In contrast, salmon fed the high VA/high Ax diet had retention values of 3.5% and 3.2% for the control group and stressed group, respectively. There was no significant interaction between diet and stress/control treatment (*p* = 0.16). There was, however, a tendency for a positive effect of high VA in the diet on astaxanthin retention in stressed salmon (*p* = 0.06), and there was also a positive effect of 60 mg astaxanthin in the diet (*p*  < 0.05) on retention during stress. For the control group, there was no significant effect of vitamin A in the diet on astaxanthin retention.

Stress did not affect the metabolism of astaxanthin to idoxanthin, and there was no significant effect of astaxanthin and VA in the diet on muscle idoxanthin concentrations (Figure 7C,D). There was, however, a significant interaction between dietary astaxanthin and VA concentration on muscle idoxanthin concentrations (*p*  < 0.05). In salmon fed a low VA diet, idoxanthin was higher in fish fed the diet with 30 ppm astaxanthin, whereas in salmon fed the high VA diet, 60 ppm astaxanthin resulted in a higher idoxanthin concentration in the muscle.

### 3.3. Carotenoid Composition Obtained by NMR

**Figure 9:**
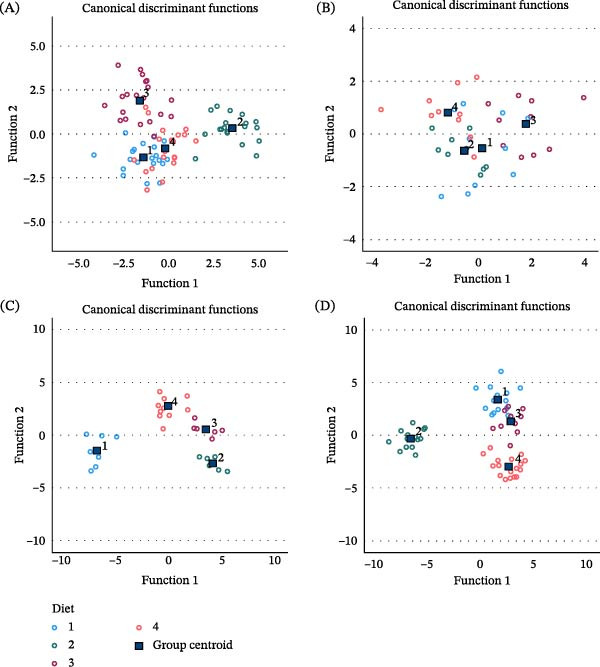
LDA plot of samples from muscle from controls (A) and stressed Atlantic salmon (B) after the 5‐week stress‐challenge phase fed four selected diets (A, C, D, F) Half of the tanks underwent thrice‐weekly low‐water/low‐oxygen challenges and half served as non‐stressed controls. *N* = 8 individuals per treatment. LDA (C) and (D) show LDA of controls and stressed samples, respectively, without “outliers”.

Dietary effects on the carotenoid profile of control fish were assessed using 123 significant variables. LDA showed good separation of samples according to diet (Figure 9A). The most distinct clustering was observed between Diet C (low astaxanthin, high VA) and Diet D (high astaxanthin, low VA), which represent opposite combinations of VA and carotenoid levels.

The same analytical approach applied to stressed fish revealed 221 significant variables, indicating metabolic regions affected by stress exposure. However, in contrast to control fish, the LDA of stressed samples (Figure 9B) showed no clustering among dietary groups.

Visual inspection of the ^1^H NMR spectra revealed a small number of outlier samples—individual fish whose carotenoid spectra deviated from the overall group trend (Figure S1). The presence of outlier spectra indicates that individual fish differed in their carotenoid metabolic responses, particularly under stress. After outlier removal and reanalysis, the number of significant variables decreased to 77 for control fish and 202 for stressed fish. LDA plots based on these refined datasets (Figure 9C,D) showed a tighter grouping of samples. However, these results should be interpreted with caution as outlier removal may obscure genuine biological variability. Interestingly, Diet F showed no outliers in the control group and fewer outliers in stressed samples, suggesting a more uniform metabolic response among fish fed this diet.

### 3.4. Microarray

**Figure 10:**
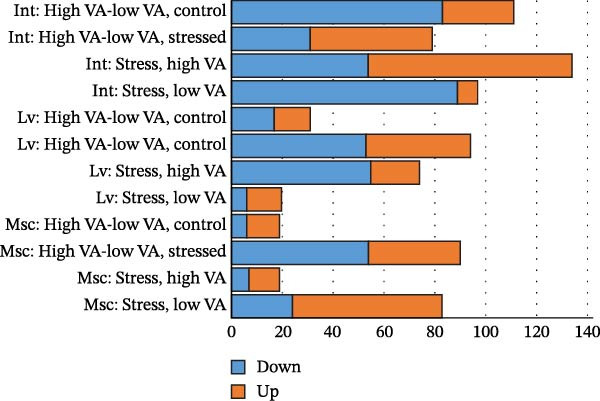
(A) The numbers of differentially expressed genes, effects of dietary VA and stress in intestine (Int), liver (Lv) and muscle (Msc) Atlantic salmon after the 5‐week stress‐challenge phase fed four selected diets (A, C, D, F). Half of the tanks underwent thrice‐weekly low‐water/low‐oxygen challenges and half served as non‐stressed controls.

The effects of dietary VA and stress on the transcriptomes were relatively small, as seen from the numbers of DEG (Figure 10). The effects were tissue‐specific, and the lists of DEG showed little overlap. Responses to VA were almost completely different in control and stressed fish, suggesting interactions between the treatments.

In the intestine, treatments affected genes with immune, metabolic, and structural functions (Figure S2). In control salmon, the effects of high VA in the diet were mainly suppressive. A large part of the downregulated genes (21 DEG) are immune genes, including seven *lectins*, mucosal proteins, and markers of B (*ig*) and T (*cd8*) cells (Figure S1). Stress reduced expression of these genes in fish fed with low VA, eliminating difference between the dietary groups. Stress also downregulated seven immune genes in the high‐VA group with no effect on fish fed with low VA. The combination of high dietary VA and stress increased the expression of several genes involved in metabolism of fatty acids and steroids, including two upregulated *cytochromes p450—cyp27c1* and *cyp26a1*. These enzymes play an important part in retinoid metabolism of mammals, producing hydroxylated forms of retinoic acid and desaturated all‐trans VA (3,4‐dehydroretinol, vitamin A_2_) [47]. Zebrafish P450 27C1 is an efficient retinol 3,4‐desaturase [48]. Stress increased expression of *apo iv* in the high VA diet group, whereas the expression pattern was opposite in control fish. High VA up‐regulated six collagens and other components of the extracellular matrix, but the effect disappeared in stressed fish.

In the liver, the combination of high VA in the diet and stress up‐regulated six chaperones (heat shock proteins and cognates) involved in responses to protein stress (Figure S3). A small number of immune genes showed different responses to VA and stress. Three complement factors were upregulated in stressed low‐VA salmon. High dietary VA increased the expression of five metabolic genes in the control and five genes in stressed salmon. The effect of stress was seen only in salmon fed with high VA: two up and six downregulated genes. *Cyp27c1* (upregulated in control high VA) is a gene of retinoid metabolism, which was also regulated in the intestine.

In skeletal muscle, high dietary VA stimulated many immune genes, and the effect was enhanced by stress: eight genes responded in both control and stressed salmon, and 15 genes showed significant differences between low and high VA only in stressed fish, though similar expression profiles were observed in nonstressed controls (Figure S4). Most of these genes encode secreted plasma proteins including acute phase proteins, complement components, and proteins of the clotting cascade. Stress upregulated three *g0/g1 switch proteins 2* associated with inflammation in the Atlantic salmon muscle [49, 50]. Combining low VA and stress increased the expression of eight metabolic genes, including four transporters. Similar effects of diet and stress were observed for *ghrelin receptor 1* and *neuropeptide y receptor y7*, involved in the regulation of energy balance.

## 4. Discussion

Astaxanthin is essential for normal development in early stages, even when the feed contains sufficient levels of VA [22–24]. Whether in vivo conversion of astaxanthin to VA has any significant biological function in seawater‐adapted salmon is not known. The content of VA in salmon diets varies considerably due to fluctuations in the raw material content. In low‐marine diets, it can be under 30,000 IU, while in a diet based on marine raw materials, it can be over 100,000 IU [26, 29, 51]. High doses of VA are toxic, and uptake in the intestine is tightly regulated by a negative feedback mechanism in mammals. Elevated levels of all‐trans retinoic acid, the active form of vitamin A1, reduce the expression of BCO1 and SR‐B1, which lowers the uptake of carotenoids and synthesis of VA [22, 23, 52–57]. If the same mechanisms exist in fish, excess dietary VA could reduce the uptake and metabolism of astaxanthin to VA.

The nutritional requirements for VA in *A. salmon* post smolts for optimal growth and survival are not well documented, but according to a study by [58], the dietary levels applied in the present study have no negative effect on growth, FCR, or liver function. The lowest dose used in the present study (6500 IU, ~2 mg/kg) was lower than concentrations tested by [58], but no significant effects of diet VA concentration were found in growth or feed utilization. The highest dietary VA concentration (100,000 IU/kg, ~30 mg/kg) reduced the astaxanthin digestibility in the present study, and both muscle retention and flesh color were also lower in salmon fed the highest VA concentration. The digestibility and retention of astaxanthin were lower at the higher dietary astaxanthin concentration. The average retention was 9.7% and 5.2% in salmon‐fed diets with 30 and 60 ppm astaxanthin, which is in accordance with results from other studies where a decreasing retention with dietary astaxanthin has been observed [1, 2, 7, 11, 59].

The astaxanthin concentration in plasma and liver was also reduced with increasing dietary VA concentrations, as in the muscle, whereas the concentration of astaxanthin in the mid‐intestine was not affected by dietary VA. Furthermore, the gene expression in the mid‐intestine of the membrane transporters *sr-b1*, *cd36*, and *abcg2* that are involved in carotenoid transport in mammals [60–62] was not affected by dietary vitamin A. But there was a downregulation of the gene *apo-a1V* in salmon fed a high VA diet in non‐stressed fish. Apolipoprotein A‐1 is the major protein in high‐density lipoproteins (HDL), which together with albumin is the main carrier of astaxanthin in salmon plasma [8, 63]. VA deficiency was found to increase the transcription of the *apoA-1* gene in rats [64], and the expression of this gene is known to be regulated by nutritional and hormonal factors. VA is likely to be involved in regulating the *apo A-I* gene expression because in vitro studies have shown that both RAR‐α and RXR‐α bind to the regulatory region of the *apo A-I* gene. A down‐regulation of this gene in the high VA diets could explain the observed reduction of astaxanthin in plasma, liver, and muscle and the lack of response in the intestine.

In vivo studies with force feeding of radioactively labeled astaxanthin and canthaxanthin show high concentration of radioactivity in bile of *A. salmon* and rainbow trout but also the intestinal epithelium had a high content of radioactivity [65–67]. Both organs have a high gene expression of the carotenoid cleaving enzymes *bco1* and β‐carotene 9^′^, 10^′^‐oxygenase (*bco2*) [68, 69]. In hepatocytes and enterocytes isolated from salmon fed the six experimental diets, idoxanthin, β‐carotene retinol, and retinal were identified as metabolites of astaxanthin. The strong positive correlations between β‐carotene and retinal suggest that β‐carotene is a precursor for retinol and is the substrate for BCO1 in salmon, as in mammals, where this enzyme has a high specificity for β‐carotene [70, 71].

Astaxanthin metabolites accounted for a larger proportion of the total radioactivity in enterocytes (40%) than that in hepatocytes (30%). It was mainly the content of idoxanthin that was higher in the enterocytes, whereas those of retinol and retinal were similar. Astaxanthin also reacts spontaneously with oxygen, producing a range of apoastaxanthinals [72]. Such metabolites were not identified with the methods used in the present study, but they could be part of the radioactive fraction with unidentified compounds. This fraction was larger in enterocytes (51% of total radioactivity) than in hepatocytes (39% of total radioactivity). In sum, our results may indicate a slightly higher metabolic turnover of astaxanthin to retinal in the intestine than in the liver. However, the liver is a large organ and plays a significant role in carotenoid clearance [73], and a larger amount of astaxanthin may metabolize in the liver than in the intestine.

Enterocytes from salmon fed the medium VA diet had the highest concentration of astaxanthin, retinol, and retinal and the lowest concentration of idoxanthin. This diet group also had the highest muscle concentration and retention of astaxanthin. The concentration of metabolites in the intestine will be influenced by the metabolic turnover in the cells but also by uptake and excretion of astaxanthin from the cells. All of these processes may potentially be affected by the dietary VA concentration. In rats and chickens, there is a dose‐dependent reduction in BCO1 activity in intestine but not in liver after oral administration of different doses of retinyl acetate [52, 53]. In rainbow trout, synthesis of VA was only detected in VA‐depleted fish [21, 74]. Thus, a higher activity and expression of *bco1* can be expected in the intestine of VA‐depleted salmon. However, no effects of dietary VA concentration were found on the expression of *bco1*, *sr-b1*, or other genes involved in retinoid transport and synthesis in the intestine. There were also no signs of deficiency or toxic effects in terms of reduced growth or increased mortality (which are not very accurate measures of nutritional requirements). According to the findings of [58], a diet concentration of 100,000 IU/kg, which was the highest level in the present study, should not induce toxic effects in *A. salmon* post smolts. There is not much available information on the minimum requirements of VA in *A. salmon* post smolts. Thompson et al. [75] reported that diet inclusion of 6500 and 50,000 IU/kg of VA improved phagocyte respiratory burst, bactericidal activity, lymphocyte functions, serum lysozyme activity, and complement activity in Atlantic salmon compared to a low VA diet (1233 IU/kg). In coho salmon (*Oncorhynchus kisutch*) post smolts, a dietary level of 6400 IU was found to be optimal for growth and activity of antioxidant enzymes in the liver [76]. The minimum level recommended by National Research Council [32] for *A. salmon* is 2500 IU/kg, but Liu et al. [26] suggested to increase the recommendation to 4000–8000 IU/kg for optimal performance when salmon are fed plant‐based diets.

One of the major challenges faced by the animal agriculture industry is the negative impact of oxidative stress on livestock health and performance [77]. Stress has been discussed as a possible cause of poor pigmentation in salmon, particularly in connection with handling and delousing operations. Data from commercial productions showed a negative correlation between fillet color and the number of mechanical delousing operations the fish had undergone in the sea phase [13]. Thermal stress also has a negative impact on the fillet color of salmon. Sea temperatures above 18–25°C for extended periods reduced the feed intake, fillet astaxanthin concentration, and color [14, 15]. In addition to the lower astaxanthin content, Vo et al. [15] also found a decrease in the content of long‐chain n‐3 fatty acids in the muscle and plasma of pale fish. There were also differences in the metabolic profiles in the muscle, intestine, and liver of salmon with pale fillets and salmon with redder fillets. The retinoid synthesis pathway was upregulated in pale fish, suggesting an interaction between the synthesis of VA and thermal stress [15]. In the present study, repeated stress in the form of crowding and hypoxia reduced the concentration and retention of astaxanthin in the fillet without affecting feed intake and growth. The muscle retention of astaxanthin was on average 3.4% in the control group and 1.4% in stressed salmon. The muscle retention of astaxanthin in salmon is dependent on dietary, environmental, and physiological factors, and retention values are typically between 5% and 10% in larger salmon fed diet concentrations around 50 ppm [3, 4, 7, 11, 12]. As mentioned previously, astaxanthin retention is negatively correlated with the dietary dose. However, in stressed salmon, a higher retention was found in salmon fed diets with 60 ppm astaxanthin, both with high and low VA concentrations. Thus, the negative effect of stress on retention was counteracted in salmon fed high doses of VA and astaxanthin, which improved flesh pigmentation during stress. The analysis with NMR showed that there was variation between individual fish in the carotenoid spectrum, which probably reflects genetic differences, sex differences, or possibly differences in health status. The NMR analyses also indicated that stress‐induced changes in carotenoid profiles were diet‐dependent and that astaxanthin metabolism was significantly affected in fish fed more VA. The variation between individual fish increased during stress, and the separation between diets was less clear than in the group that was not stressed. Interestingly, the high‐VA/high‐astaxanthin diet showed no outliers in the control group and fewer outliers in stressed fish, suggesting a more uniform metabolic response among fish fed this diet.

Unfortunately, enterocytes and hepatocytes were isolated at the end of the 17‐week feeding trial from fish that had not been subjected to stress, so the potential effects of stress on the concentration of astaxanthin metabolites in these cell types could not be assessed. This should be a task for future work on the effects of stress on the astaxanthin metabolism.

Stress had several effects on gene expression in the examined tissues. In the mid‐intestine of salmon that received high VA in the feed, stress increased the expression of several genes involved in the metabolism of fatty acids and steroids, while in the liver, there was an upregulation of heat shock proteins. In muscle, high VA stimulated many immune‐related genes, and stress further enhanced this effect. The low VA content in the diet of stressed salmon increased the expression of eight metabolic genes, including four ion transporters. However, there were no effects of stress or high and low dietary VA on the expression of genes in the retinoid pathway or in the oxidative stress‐response pathways.

One gene that was expressed differently in stressed salmon and controls was *apo-a1V*, which is the major protein in HDL, the main carrier of astaxanthin in salmon plasma. In salmon fed high‐VA diets, this gene was downregulated in controls but upregulated in stressed salmon. As mentioned previously, the expression of this gene is regulated by nutritional and hormonal factors in mammals, and VA probably affects its expression because both RAR‐α and RXR‐α bind to the regulatory region of the apo A‐I gene. The mechanisms behind the observed effects may be a result of the regulatory effects of VA derivatives (retinoids) on metabolic enzymes involved in carotenoid breakdown. Negative feedback regulating the breakdown of astaxanthin at high dietary VA levels can explain the lower pigmentation efficiency. Medium‐dietary VA is sufficient to avoid deficiency without strongly triggering the induction of catabolic enzymes or competing pathways. Thus, enzyme activity and gene expression remain balanced, resulting in lower astaxanthin breakdown and greater retention in the muscle. Both VA and carotenoids like astaxanthin use some of the same transporters and metabolic pathways in the intestine and liver. When VA levels are high, the body may prioritize VA metabolism, increasing the flux through shared or overlapping enzymatic steps, leading to greater astaxanthin degradation and reduced uptake in the intestine. Stress could also induce the *in vivo* synthesis of VA, as was found in heat‐stressed Atlantic salmon in Tasmania. If the diet contained more VA and astaxanthin, this could be beneficial during stress because it could reduce the salmon’s need for synthesizing VA from astaxanthin and leave more available for fillet deposition. However, the mechanisms for the uptake and metabolism of astaxanthin are not fully understood in salmon, and more work is required within this field to explain the findings in this trial.

## 5. Conclusion

In conclusion, when salmon was not exposed to stress, the highest dose of VA in the diet (100,000 IU/kg) had a negative impact on fillet color, astaxanthin digestibility, and retention, whereas a medium concentration of VA (35,000 IU) was optimal for fillet pigmentation. Stress had a negative effect on fillet astaxanthin concentration and retention. Within the dietary combinations tested during the stress challenge, the combination of high doses of vitamin A and astaxanthin was associated with a reduced loss of fillet pigmentation in stressed salmon.

However, as the medium VA/high astaxanthin diet was not included in the stress experiment, it cannot be concluded whether moderate VA levels combined with high astaxanthin would also mitigate stress‐induced pigment loss. The stress challenge was also performed in duplicate tanks, which could have reduced the statistical power of the trial. Further research is needed to clarify this and to optimize feed formulations with respect to antioxidant concentrations that may improve the fillet quality in salmon that experience stress. The scale and character of gene expression changes found in the present trial are common for feed trials, which do not cause large differences between the study groups. We found consistent expression changes in three tissues, which are most likely too small to affect the health and performance of salmon. Concerning individual genes, of note were two *cytochromes p450*, which are most likely involved in retinoid metabolism and responded to high VA levels.

## Funding

This work was supported by the Norwegian Seafood Research Fund (FHF, Project 901623) and Skretting Aquaculture Innovation.

## Ethics Statement

All experimental procedures were conducted according to the guidelines of the Norwegian State Commission for Laboratory Animals. The protocol was approved by the National Food Safety Authority (Identification Number: FOTS ID 24308).

## Conflicts of Interest

The authors declare no conflicts of interest.

## Supporting Information

Additional supporting information can be found online in the Supporting Information section.

## Supporting information


**Supporting Information** Table S1: Idoxanthin and total carotenoid concentrations in plasma, intestine and liver in A. salmon fed six diets with different concentrations of vitamin A (Low, Medium, High) and two concentrations of astaxanthin (30 or 60 mg/kg) for 17 weeks. Values are mean ± SEM (*N* = 3). Figure S1: Carotenoid region of 1H NMR spectra of salmon muscles samples from diet A. Green arrows showing the detected outlier signals. Figure S2: Genes with differential expression in the mid‐intestine. Data are folds to the mean of the entire data set. Column VA shows significant dietary effects in control (C) and stressed (S) groups. Column Str shows significant effect of stress in groups fed with high (H) and low (L) levels of retinol. Figure S3: Genes with differential expressions in the liver. Data are folds to the mean of the entire data set. Column VA shows significant dietary effects in control (C) and stressed (S) groups. Column Str shows significant effect of stress in groups fed with high (H) and low (L) levels of retinol. Figure S4: Genes with differential expression in skeletal muscle. Data are folds to the mean of the entire data set. Column VA shows significant dietary effects in control (C) and stressed (S) groups. Column Str shows significant effect of stress in groups fed with high (H) and low (L) levels of retinol.

## Data Availability

Data will be made available upon request.
