# Supplementary material for: Effects of Dietary Vitamin A Concentration and Stress on Astaxanthin Utilization in Atlantic Salmon (Salmo salar)
Source: Aquac Nutr. 2026 Jul 14;2026:1961029. doi: 10.1155/anu/1961029 (PMC13369587; doi:10.1155/anu/1961029)
Supplement: Supplementary file 1 — Supporting Information Table S1: Idoxanthin and total carotenoid concentrations in plasma, intestine and liver in A. salmon fed six diets with different concentrations of vitamin A (Low, Medium, High) and two concentrations of astaxanthin (30 or 60 mg/kg) for 17 weeks. Values are mean ± SEM (N = 3). Figure S1: Carotenoid region of 1H NMR spectra of salmon muscles samples from diet A. Green arrows showing the detected outlier signals. Figure S2: Genes with differential expression in the mid‐intestine. Data are folds to the mean of the entire data set. Column VA shows significant dietary effects in control (C) and stressed (S) groups. Column Str shows significant effect of stress in groups fed with high (H) and low (L) levels of retinol. Figure S3: Genes with differential expressions in the liver. Data are folds to the mean of the entire data set. Column VA shows significant dietary effects in control (C) and stressed (S) groups. Column Str shows significant effect of stress in groups fed with high (H) and low (L) levels of retinol. Figure S4: Genes with differential expression in skeletal muscle. Data are folds to the mean of the entire data set. Column VA shows significant dietary effects in control (C) and stressed (S) groups. Column Str shows significant effect of stress in groups fed with high (H) and low (L) levels of retinol. [file ANU-2026-1961029-s001.docx]

**Supplements**

Table S1: Idoxanthin and total carotenoid concentrations in plasma, intestine and liver in A. salmon fed six diets with different concentrations of vitamin A (Low, Medium, High) and two concentrations of astaxanthin (30 or 60 mg/kg) for 17 weeks. Values are mean ± SEM (N= 3)

|  |  | Diet A  30/L | Diet B  30/M | Diet C  30/H | Diet D  60/L | Diet E  60/M | Diet F  60/H | 1-Way ANOVA |
| --- | --- | --- | --- | --- | --- | --- | --- | --- |
| Idoxanthin | plasma | 0.4±0.0 | 0.3±0.1 | 0.3±0.1 | 0.4±0.1 | 0.4±0.1 | 0.3±0.0 | P=0.75 |
| (mg/kg) | mid-intestine | 0.2±0.0 | 0.1±0.0 | 0.1±0.0 | 0.2±0.0 | 0.2±0.1 | 0.2±0.0 | P=0.53 |
|  | liver | 0.2±0.0 | 0.1±0.0 | 0.1±0.0 | 0.3±0.1 | 0.2±0.0 | 0.1±0.0 | P=0.09 |
| Total carotenoids | plasma | 2.9±0.1^ab^ | 2.7±0.0^ab^ | 2.4±0.2^b^ | 3.2±0.2^a^ | 2.8±0.1^ab^ | 2.6±0.0^b^ | P<0.01 |
| (mg/kg) | mid-intestine | 0.9±0.2 | 0.8±0.0 | 1.0±0.2 | 1.5±0.0 | 1.6±0.3 | 1.4±0.2 | P=0.08 |
|  | liver | 1.4±0.1^bc^ | 1.3±0.1^bc^ | 1.0±0.1^c^ | 2.3±0.3^a^ | 1.8±0.1^ab^ | 1.4±0.1^bc^ | P<0.001 |
| Idoxanthin | plasma | 13.8±0.3 | 12.5±2.5 | 13.1±2.8 | 13.2±1.8 | 13.3±3.5 | 11.7±1.7 | P=0.98 |
| (% of carotenoids) | mid-intestine | 19.6±1.3 | 16.1±2.7 | 12.7±0.6 | 14.7±2.9 | 13.7±1.7 | 12.2±2.1 | P=0.19 |
|  | liver | 13.3±0.8 | 11.8±1.4 | 9.1±1.2 | 11.6±2.0 | 12.3±2.9 | 9.5±1.7 | P=0.53 |

**
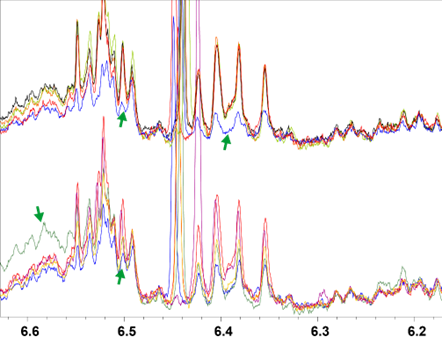
**

**Figure S1.** Carotenoid region of 1H NMR spectra of salmon muscles samples from diet A. Green arrows showing the detected outlier signals.

**
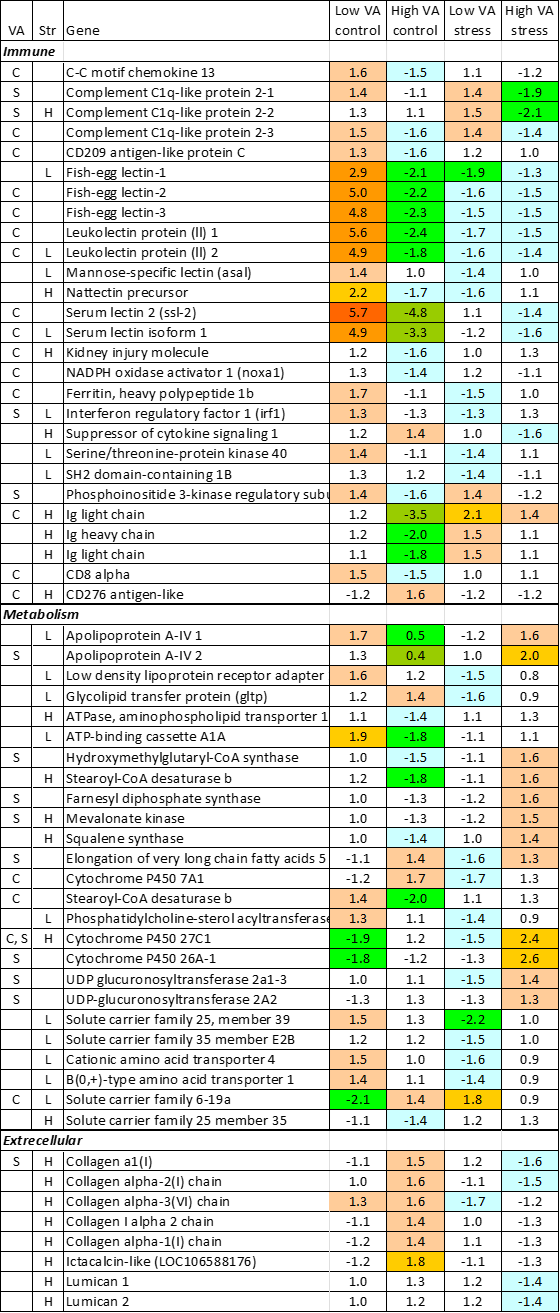
**

**Figure S2**. Genes with differential expression in the mid-intestine. Data are folds to the mean of the entire data set. Column VA shows significant dietary effects in control (C) and stressed (S) groups. Column Str shows significant effect of stress in groups fed with high (H) and low (L) levels of retinol.

**
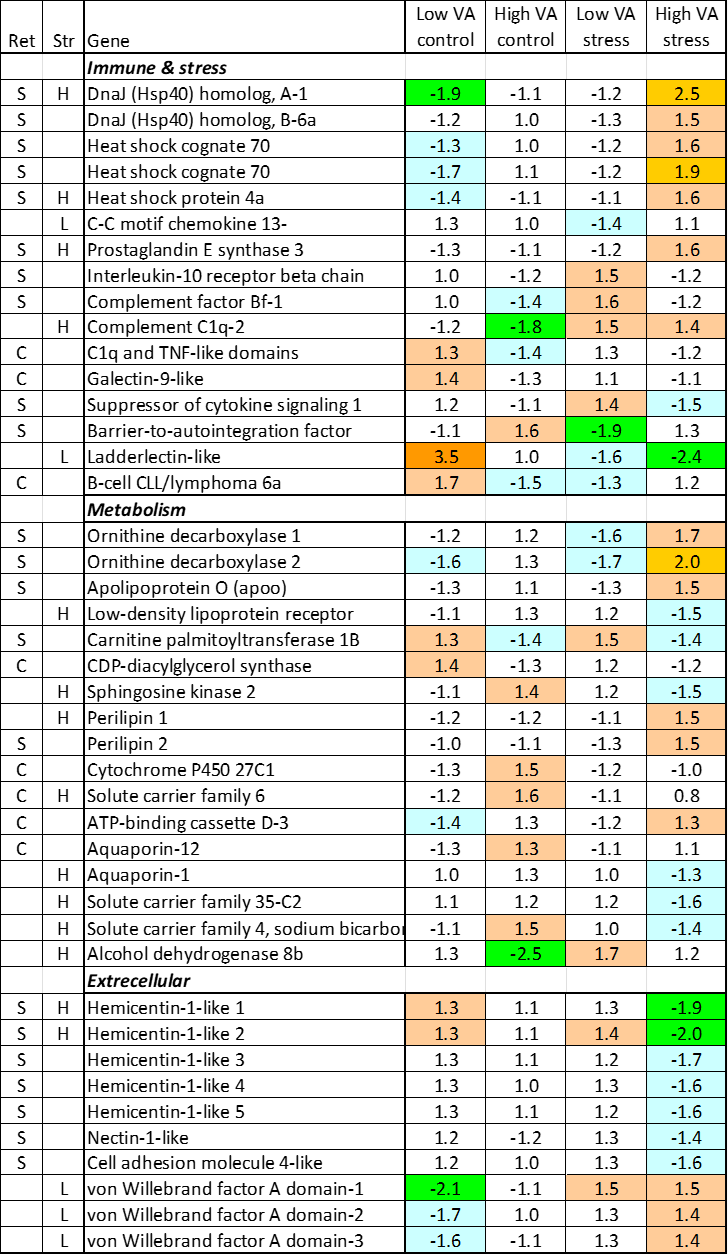
**

**Figure S3:** Genes with differential expressions in the liver. Data are folds to the mean of the entire data set. Column VA shows significant dietary effects in control (C) and stressed (S) groups. Column Str shows significant effect of stress in groups fed with high (H) and low (L) levels of retinol.


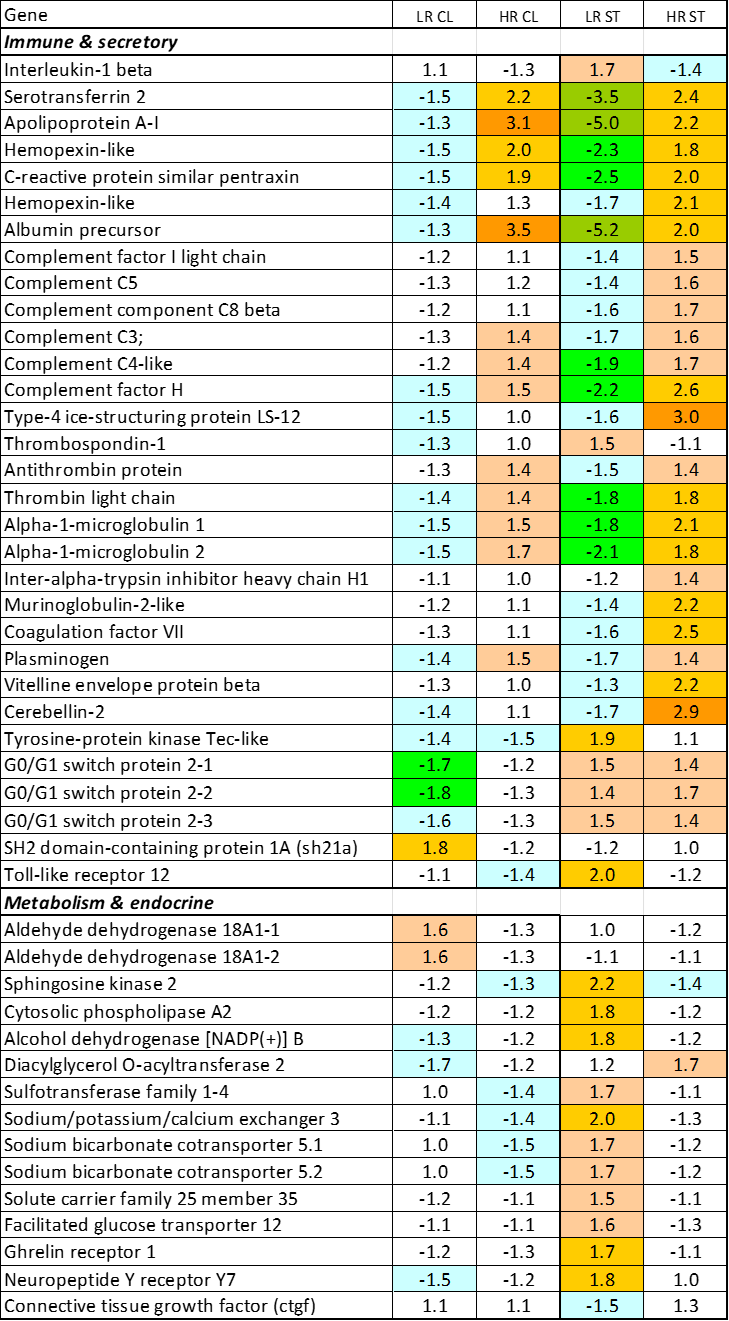

**Figure S4:** Genes with differential expression in skeletal muscle. Data are folds to the mean of the entire data set. Column VA shows significant dietary effects in control (C) and stressed (S) groups. Column Str shows significant effect of stress in groups fed with high (H) and low (L) levels of retinol
